# First experimental feasibility study of VIPIC: a custom-made detector for X-ray speckle measurements

**DOI:** 10.1107/S1600577516000114

**Published:** 2016-02-10

**Authors:** Abdul K. Rumaiz, D. Peter Siddons, Grzegorz Deptuch, Piotr Maj, Anthony J. Kuczewski, Gabriella A. Carini, Suresh Narayanan, Eric M. Dufresne, Alec Sandy, Robert Bradford, Andrei Fluerasu, Mark Sutton

**Affiliations:** a Photon Science Directorate, Brookhaven National Laboratory, Upton, NY 11973, USA; b Fermi National Laboratory, Batavia, IL 60510, USA; c Department of Metrology and Electronics, AGH University of Science and Technology, Krakow, Poland; d SLAC National Accelerator Laboratory, Menlo Park, CA 94025, USA; e X-ray Science Division, Advanced Photon Source, Argonne National Laboratory, Argonne, IL 60439, USA; f Physics Department, McGill University, Montreal, Quebec, Canada H3A2T8

**Keywords:** VIPIC, XPCS, detectors

## Abstract

The Vertically Integrated Photon Imaging Chip (VIPIC) was custom-designed for X-ray photon correlation spectroscopy, an application in which occupancy per pixel is low but high time resolution is needed. VIPIC operates in a sparsified streaming mode in which each detected photon is immediately read out as a time- and position-stamped event. This event stream can be fed directly to an autocorrelation engine or accumulated to form a conventional image. The detector only delivers non-zero data (sparsified readout), greatly reducing the communications overhead typical of conventional frame-oriented detectors such as charge-coupled devices or conventional hybrid pixel detectors. This feature allows *continuous* acquisition of data with timescales from microseconds to hours. In this work VIPIC has been used to measure X-ray photon correlation spectroscopy data on polystyrene latex nano-colliodal suspensions in glycerol and on colloidal suspensions of silica spheres in water. Relaxation times of the nano-colloids have been measured for different temperatures. These results demonstrate that VIPIC can operate *continuously* in the microsecond time frame, while at the same time probing longer timescales.

## Abstract 2

Preliminary X-ray correlation spectroscopy results from the novel three-dimensional vertically integrated photon imaging chip (VIPIC) detector are presented.

## 1. Introduction   

X-ray correlation spectroscopy (XCS) has gained importance as a powerful tool for the study of dynamics primarily in soft matter and colloids (Dierker *et al.*, 1995 ▸; Lu *et al.*, 2010 ▸; Lurio *et al.*, 2000 ▸; Patel *et al.*, 2006 ▸). Key to the success of this technique is the availability of highly coherent X-ray beams produced by third-generation synchrotron sources. In this technique the fluctuation in the coherent scattered intensity can be used to interpret the dynamics of the material. Using X-rays provides a significant advantage with regard to the length scale of the system being probed (when compared with visible-light-based dynamic light scattering). However, the time scale that can currently be probed with this technique is currently limited (Thurn-Albertch *et al.*, 1996 ▸). Several modern detectors have demonstrated frame readout rates in the kHz and even MHz range (Hatsui & Graafsma, 2015 ▸). However, most of these detectors cannot sustain these rates indefinitely or continuously. This limitation is mainly driven by the data rate resulting from non-sparsified readout. In this work we present preliminary XCS results from our novel three-dimensional vertically integrated photon imaging chip (VIPIC).

## 2. Detector   

**Figure 1:**
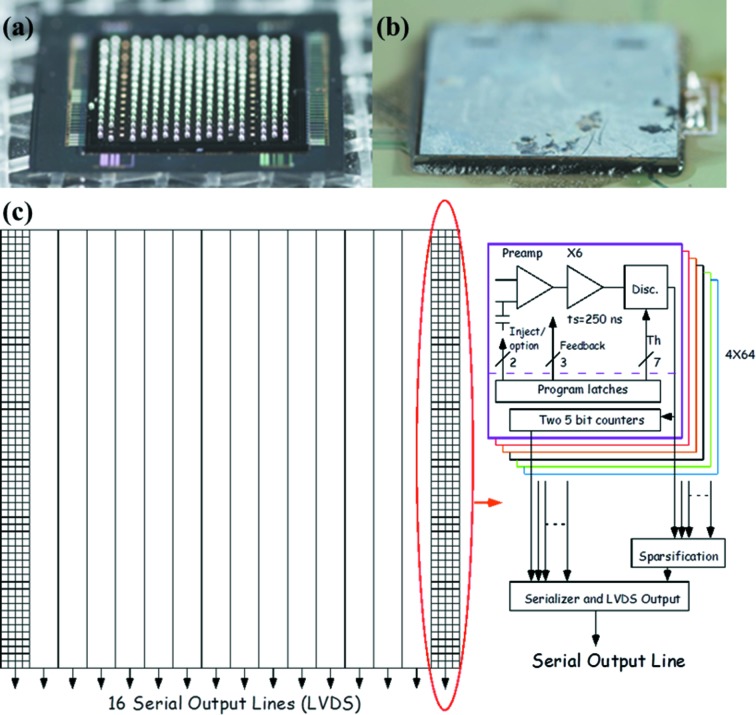
Images of the VIPIC + sensor die. (*a*) After deposition of solder bumps onto the back of an ASIC. (*b*) After flip-chip bonding of VIPIC + sensor to the PCB. (*c*) VIPIC organization. The dashed line in the box on the right-hand side indicates the split between the different CMOS layers. The prototype is a matrix of 64 × 64 pixels, which are divided into 16 groups. Each group has one serializer and low-voltage differential signaling (LVDS) driver that allows outputting the data from the group.

The design details and test results of the VIPIC chip can be found elsewhere (Deptuch *et al.*, 2014 ▸). To summarize, the VIPIC project takes advantage of the three-dimensional integration technology which is now actively pursued by the microelectronic industry for high-performance next-generation integrated circuits (ICs) (Kim *et al.*, 2009 ▸; Lee & Chakrabarty, 2009 ▸). The VIPIC chip was designed in the Global Foundries 130 nm process with silicon vias embedded right after front-end-of-line processing. The integration of different tiers (analog, digital and the sensor) was achieved by Cu–Cu thermo-compression or Cu-based oxide–oxide bonding. The chip is a 64 × 64 pixel device with a pitch of 80 µm, making the total active area 5120 µm × 5120 µm. Figs. 1(*a*) and 1(*b*) ▸ show the VIPIC die mounted on a printed circuit board (PCB) for bench testing. The unique feature of the chip is the priority encoder based sparsification circuitry. Only pixels that are hit are read out, thus greatly reducing the volume of data to be transmitted off-chip. The priority encoder scheme guarantees that the time to signal an event is independent of the position of the hit in the detector.

Fig. 1(*c*) ▸ shows the VIPIC organization. The charges from the sensor are integrated in a conventional charge-sensitive amplifier (CSA), and then filtered in a shaping amplifier. The CSA has an adjustable feedback resistor to accommodate a range of sensor leakage currents. The shaped pulse is passed to a discriminator with adjustable threshold. If the pulse exceeds the threshold, a hit is signaled to the digital section. The digital section records the arrival of hits, accumulating multiple hits in one of a pair of 5-bit counters in each pixel. The logic causes one counter to be accumulating while the other is being read out, so there is no dead-time due to readout, and all events recognized by the analog section are registered. The pixel hits are fed into a priority encoder circuit which generates the address of the highest pixel with a hit. This is then read out, including the counter contents, and the hit is then reset. The next highest hit is then made active and is read out. This cycle proceeds until all hits are read out, or the next time-slice clock arrives. The time-slice clock period determines the time resolution of the experiment, and can be set to any value above 150 ns. This period places an upper limit on the number of hits in a 256-pixel slice, since the readout is serial, with a 100 MHz clock. In practice this should not be a problem, since, for a 10 µs time slice, this represents an incident flux of roughly 4 × 10^8^ photons s^−1^ cm^−2^, which is very high for photon correlation experiments.

## 3. Experiment   

Two samples were prepared to demonstrate the broad capabilities of the detector. The first was an aqueous solution of 70 nm polystyrene particles dispersed in glycerol. The second was a colloidal suspension of 150 nm silica spheres in water. In both cases the solution was sealed in a capillary and vacuum checked prior to loading. The X-ray scattering experiments were performed at beamline 8-ID-I of the Advanced Photon Source (APS) at Argonne National Laboratory. The beam size was fixed at 20 µm × 20 µm and an energy of 10 keV. The XCS experiments were performed with a version of VIPIC which had its sensor also bonded to the two-tier ASIC using an oxide-bonding technique. This technology provides significantly reduced noise compared with bump-bonding (Deptuch *et al.*, 2014 ▸). The time base for acquisition was 3.6 µs, and the data were collected in sequences of 3.5 × 10^8^ periods corresponding to around 20 min each.

The detailed theory relevant to XCS measurements can be found elsewhere (Fluerasu *et al.*, 2008 ▸, 2010 ▸; Busch *et al.*, 2008 ▸; Narayanan *et al.*, 1997 ▸). Briefly, the experiment involves measuring the intensity autocorrelation function given by
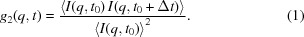
This is related to the intermediate scattering function *g*_1_(*q*,*t*) *via* the Siegert relation given by
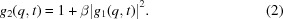
β is the contrast or visibility factor and depends on coherence and scattering geometry. For the simple case of particle diffusion in liquids *g*_1_ can be 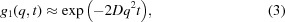
where *D* is the Stokes–Einstein diffusivity of the particle. In this work we collected XCS data for a polystyrene suspension in glycerol at different temperatures. To gain improved signal-to-noise ratio we collected several data sets under identical conditions. Within a data set the intensity autocorrelation was computed for each pixel and then averaged over desired *q* bins.

## 4. Detector characterization   

**Figure 2:**
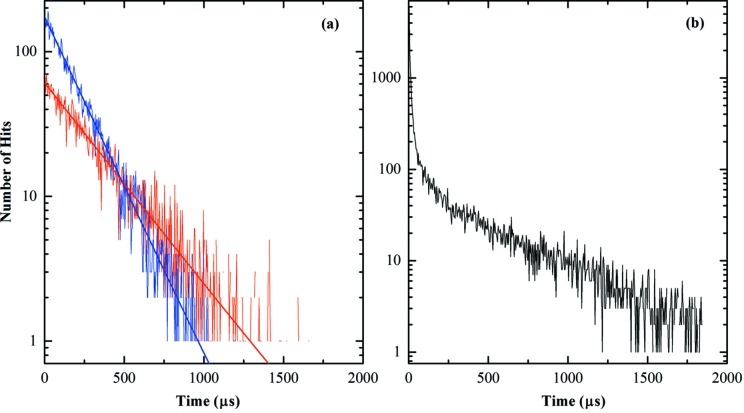
(*a*) Arrival statistics of three ‘good pixels’ plotted on a semi-log scale with the exponential fit. (*b*) Arrival statistics of a ‘bad pixel’. The discontinuity in the slope at short times is obvious.

In the following we will first discuss the measurements on the latex sample. We begin with an analysis of photon arrival statistics in the VIPIC detector in order to verify that the detector is performing as expected, and not distorting the data. We define a ‘hit’ to imply an X-ray photon interacting with the detector such as to exceed the detection threshold. If the incident X-ray beam has a Poissonian character, then the temporal distribution of detected events should also be Poissonian if the detector is accurately detecting those photons. To test this, we analyzed the event data looking for the number of times we saw consecutive hits to the same pixel, spaced δ*t* apart, with δ*t* ranging from one time-stamp interval (3.6 µs for this run) to a few milliseconds. If the detection process is Poissonian, a histogram of these numbers *versus* time interval should decay exponentially (Yu & Fessler, 2000 ▸). Fig. 2 ▸ shows such a histogram. The *x*-axis is the time between photons in a given pixel and the *y* axis is the number of occurrences with that time elapsed since the previous event in that pixel (on a logarithmic scale). Fig. 2(*a*) ▸ shows the data from one pixel. The slope of this straight line depends on the average intensity in that pixel, becoming steeper as the intensity increases. It is clear that the data fall on a logarithmic straight line, *i.e.* an exponential curve (fitting the curve with exponential fit over the entire range gives a value of *R* ^2^ greater than 0.94). This is consistent with a Poisson arrival distribution. If a pixel is suffering from excess noise for some reason, the histogram typically shows a non-linear behavior at short times. Fig. 2(*b*) ▸ shows data from a bad pixel. The non-linearity of the plot at short times is obvious. Inclusion of such noisy data in the correlation analysis would distort the result and so was excluded.

**Figure 3:**
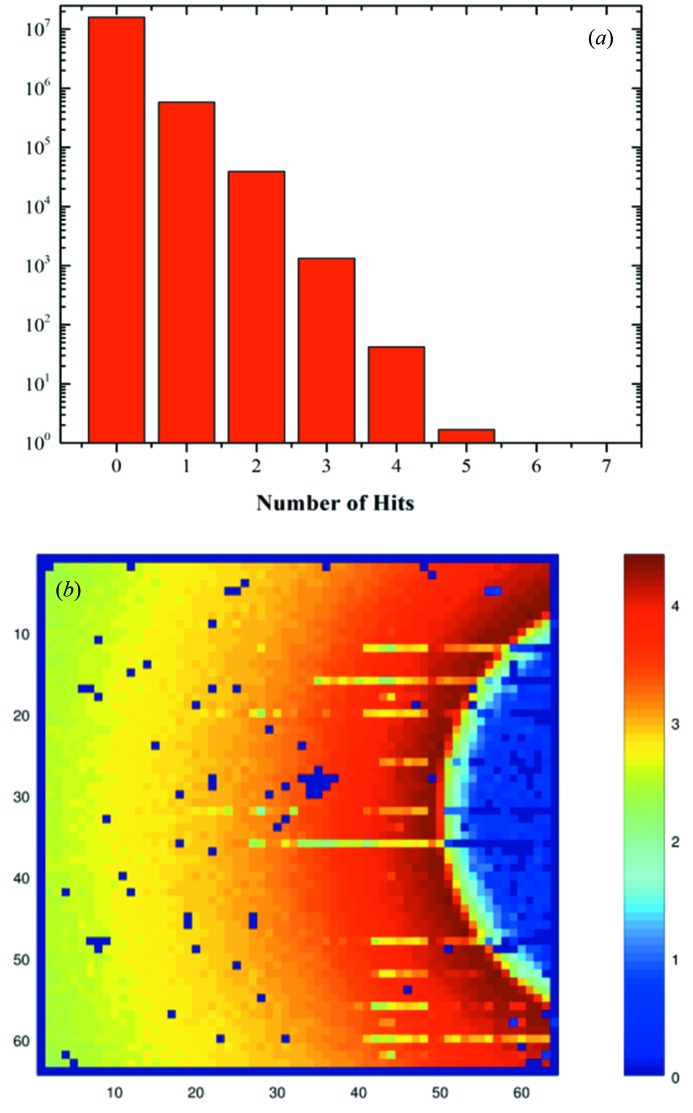
(*a*) Histogram, on a logarithmic scale, of the number of hits in one serial stream within one integration frame. (*b*) The accumulated intensity (logarithmic intensity scale). The number of dead pixels was about 5%.

The sparsified readout provides the biggest advantage when the number of hits per time base is small. Readout of each VIPIC event requires a time of order 100 ns. This suggests that, for a 3.6 µs time base, 36 events can be read out per time base per readout channel. Readout of the chip occurs over a set of serial data links, each of which handles data from 256 pixels. Sixteen of these channels make up the full 4096-pixel detector readout. The maximum occupancy at a 3.6 µs clock rate is therefore 36/256, or 14%. If this is the case, most of the data read by a conventional system would be zeros. We note that, if a longer time base clock is adequate for the system under study, the allowed occupancy increases, such that it reaches 100% for a clock period of 25.6 µs. At this point the argument for sparsification fails since there are no null data. In fact VIPIC is capable of detecting multiple hits (up to 32) in a pixel within a time frame, and the time to read out this multi-hit data is the same as that for the single-hit data, so the effect is to have an image frame rate of 39 kHz, with a maximum rate capability of 1.25 MHz per pixel. The properties of the beamline and the sample did not provide high enough intensities for this to be needed in this experiment. However, we did need to check to be sure that this was indeed the regime we were working in. Fig. 3(*a*) ▸ shows a histogram (on a log scale) of the number of hits from one serial stream (*i.e.* 256 pixels) within one integration frame. It is evident from the histogram that we only observe intensities of five hits per frame or less. The vast majority of time frames have either zero or one hit per 256 pixels per frame. Fig. 3(*b*) ▸ shows the integrated intensity image, *i.e.* the time-averaged scattering pattern, generated by summing all of the hits in each pixel over the data from which all of the results presented here were taken. There are several pixels which are excluded from the data analysis, and show as dark squares on the image. Since the experiment is not an imaging experiment, these missing pixels are not scientifically important. We expect that, as the technology becomes more mature, the number of these imperfections will decrease. This unit showed about 5% ‘bad’ pixels.

## 5. Results   

**Figure 4:**
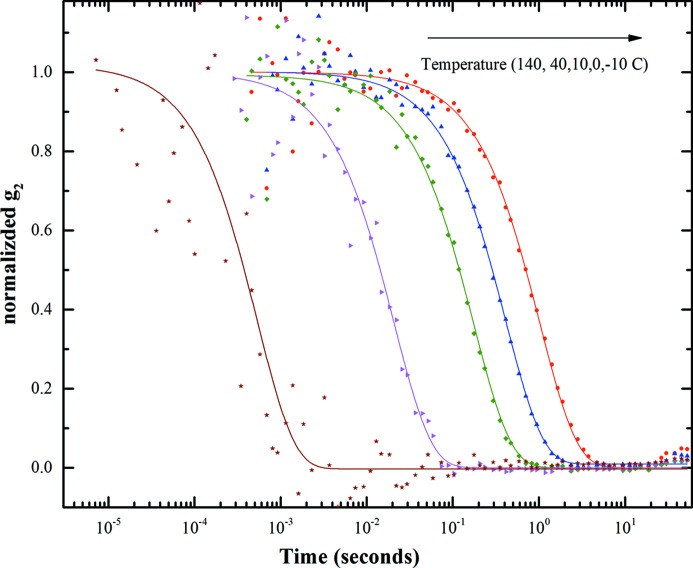
Measured intensity autocorrelation function of polystyrene particles suspended in glycerol at different temperatures.

Fig. 4 ▸ shows the average intensity autocorrelation function for spatial frequency *q* = 0.024 nm^−1^ for 70 nm-diameter polystyrene particles dispersed in glycerol for different temperatures. As mentioned before, the average intensity autocorrelation function was calculated from the per-pixel intensity weighted average of *g*_2_ in a given *q* region (for our analysis we have partitioned the data in nine *q* regions). The data shown correspond to an exposure of 20 min with 3.5 × 10^8^ frames. The number of pixels represented in the data shown corresponds to about 450 pixels. Assuming a single exponential decay, we have fit the intensity autocorrelation with equation (2). For clarity and to remove the effect of the visibility factor β, the fitted data were normalized.

**Figure 5:**
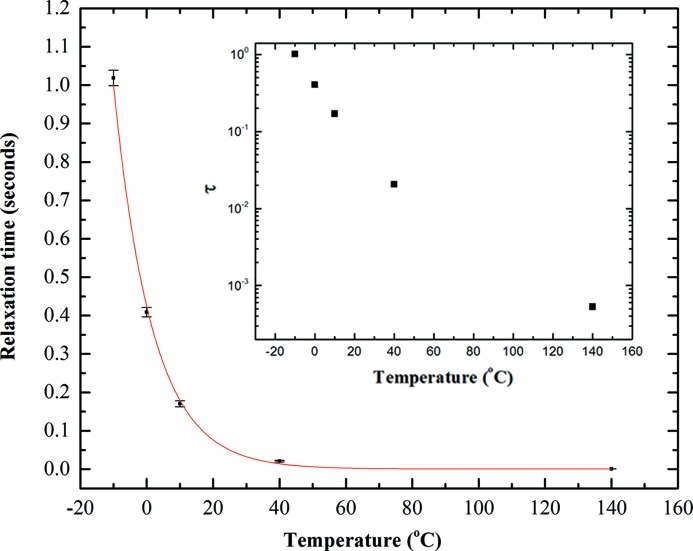
Relaxation time calculated from fitting the autocorrelation functions. The red line indicates the fit using the VFT function. The inset shows the relaxation time on a logarithmic scale.

The relaxation time (τ) calculated for the different temperatures measured are plotted in Fig. 5 ▸. It is well known that the relaxation of a colloidal suspension shows a temperature dependence well represented by the Vogel–Fulcher–Tammann (VFT) equation (Dagdug, 2000 ▸; Chen *et al.*, 2013 ▸),
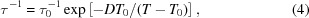
where *D* is the fragility parameter. The fitting in Fig. 5 ▸ shows reasonable agreement with the Vogel–Fulcher law. On the low-temperature (−10°C) limit the calculated relaxation time is 1.0 ± 0.2 seconds and on the high-temperature (140°C) it is 0.53 ± 0.04 milliseconds. These measurements clearly show the two strengths of the VIPIC chip: the ability to reach sub-millisecond relaxation times and the wide dynamic time range.

**Figure 6:**
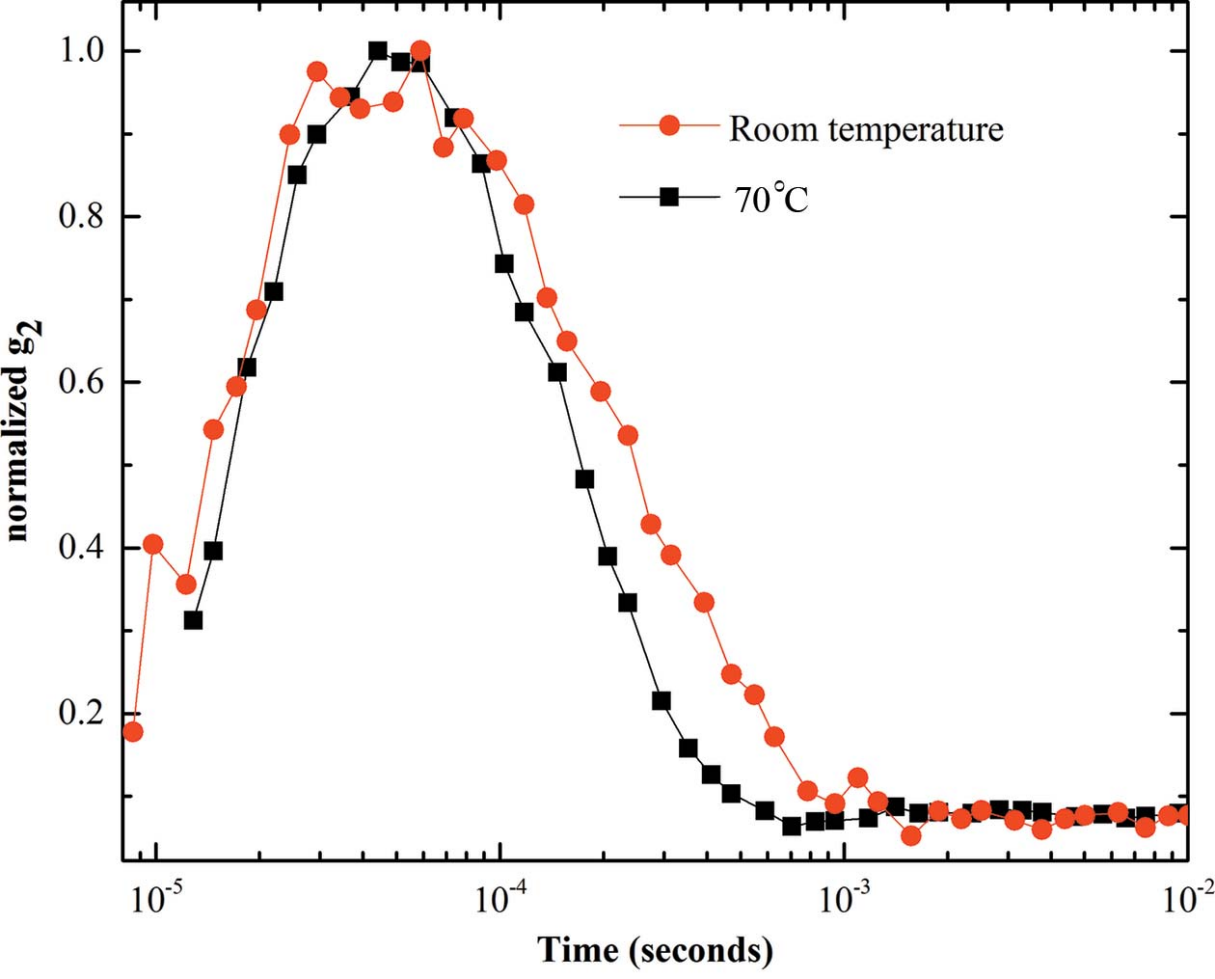
Measured intensity autocorrelation function of silica nanoparticles suspended in water at different temperatures.

We also made measurements of a sample of silica microspheres suspended in water. This sample is different from the latex sample in two ways. First, the suspension medium (water) is significantly less viscous than the glycerol solution used with the latex sample. Second, the X-ray contrast between the silica and the water is greater, providing a larger scattering signal. Unlike glycerol solutions, the viscosity of water is only weakly dependent on temperature and so we could not explore as wide a range of relaxation times as we did with the latex sample. Nevertheless, we made measurements at room temperature and 70°C, and were easily able to see a change in relaxation time. Fig. 6 ▸ shows the correlation curves for these two measurements. The much improved signal-to-noise obtained is the result of the enhanced scattering signal. The low viscosity of water pushes the relaxation times down to around 1 millisecond. Our data show low noise down to a few microseconds. The correlation fall-off at short times we attribute to beam position fluctuation in the 10 kHz range. This is certainly possible since the power supplies that drive the storage-ring magnets have ripple in this frequency range. Such high-frequency effects are typically not monitored by machine diagnostics.

**Figure 7:**
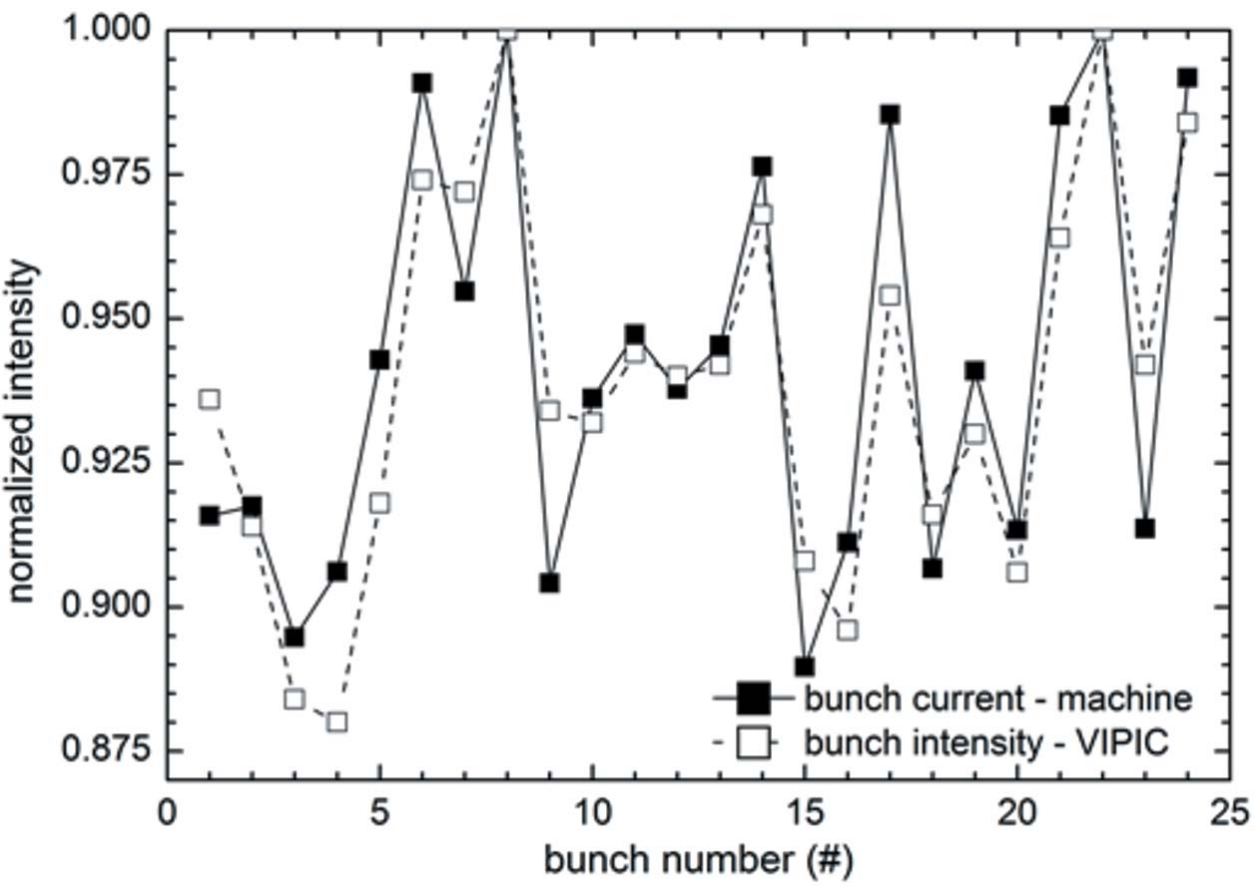
Intensity variations within the bunch filling pattern of the APS storage ring as measured by VIPIC, compared with the machine diagnostic data.

The ultimate time resolution of this detector is determined by the speed of the read clock. We were able to adjust this to 153 ns, the time between successive storage-ring electron bunches in the APS operating mode which was in use during our experiments. We synchronized this clock to the accelerator bunches. At this speed, one event can be read out per readout clock, corresponding to an overall rate of 6.5 MHz. Fig. 7 ▸ shows data collected at this speed, compared with the accelerator diagnostic readout of the bunch filling structure. It can be seen that there are bunch to bunch differences at the 10% level, and our detector result closely matches the machine diagnostic. This is a clear indication of the correct performance of the detector well down below 1 µs.

## 6. Conclusions   

We have shown XCS results using the three-dimensional integrated pixelated chip VIPIC. We have measured correlation data over the range from microseconds to seconds with this single detector, and shown sub-millisecond relaxation times. Given sufficient intensity, it is now possible to collect high-quality data down to the microsecond time scale, which opens up the XCS field to a much wider range of scientifically interesting experiments. The newest generation of synchrotron radiation sources such as NSLS-II will provide significantly enhanced coherent beam intensities, and even brighter sources are being planned. Without custom-designed detectors such as VIPIC, these new sources will not produce improved XCS data. The device presented in this paper is a prototype and only serves to demonstrate the principle and feasibility. We are currently developing a full scale (1 megapixel) version which should be available for tests in roughly two years.
